# Peracetic Acid vs. Sodium Hypochlorite: Degradation and Transformation of Drugs in Wastewater

**DOI:** 10.3390/molecules25102294

**Published:** 2020-05-13

**Authors:** Giovanni Luongo, Lucio Previtera, Afef Ladhari, Giovanni Di Fabio, Armando Zarrelli

**Affiliations:** 1 Department of Chemical Sciences, University of Napoli Federico II, Via Cintia 4, I-80126 Naples, Italy; giovanni.luongo@unina.it (G.L.); difabio@unina.it (G.D.F.); 2 Associazione Italiana per la Promozione delle Ricerche su Ambiente e Salute umana, Via Campellone 50, 82030 Dugenta (BN), Italy; previter@unina.it; 3 Laboratoire GREEN-TEAM (LR17AGR01), Université de Carthage, Institut National Agronomique de Tunisie (INAT), 43 avenue Charles Nicolle, 1082 Tunis, Tunisia; afef.ladh@yahoo.fr

**Keywords:** caffeine, tramadol, irbesartan, diclofenac, trazodone, peracetic acid, degradation byproducts, disinfection treatments

## Abstract

Numerous substances from different chemical sectors, from the pharmaceutical industry to the many consumer products available for everyday usage, can find their way into water intended for human consumption and wastewater, and can have adverse effects on the environment and human health. Thus, the disinfection process is an essential stage in water and wastewater treatment plants to destroy pathogenic microorganisms but it can form degradation byproducts. Sodium hypochlorite is the most common disinfectant, but the most important drawback associated with this kind of compound is the generation of toxic disinfection byproducts. Many studies have been carried out to identify alternative disinfectants, and in the last few years, peracetic acid has been highlighted as a feasible solution, particularly in wastewater treatment. This study compares the transformations of five emerging pollutants (caffeine, tramadol, irbesartan, diclofenac, trazodone) treated with peracetic acid, to evaluate their degradation and the possible formation of byproducts with those obtained with sodium hypochlorite. Although peracetic acid has many advantages, including a wide field of use against microorganisms and a low toxicity towards animal and plant organisms, it is not as effective in the degradation of the considered pollutants. These ones are recovered substantially and are unchanged quantitatively, producing a very low number of byproducts.

## 1. Introduction

Social, productive, and recreational activities require and use a large quantity of water [1], with the direct consequence of the production of discharges that, in order to be returned to the environment, must necessarily be purified. Urban wastewater which, in the past, almost exclusively contained biodegradable substances, currently presents greater problems of disposal due to the ever-increasing presence of chemical compounds of synthetic origin, used mainly in the industrial or pharmaceutical sector [2,3]. Seas, rivers and lakes are not able to receive a quantity of polluting substances higher than their own self-purifying capacity without seeing the water quality deteriorate and the normal balance of the ecosystem compromised. The need to purify wastewater through various treatment systems is therefore evident [4]. Regardless of the need for purification processes in terms of environmental impact, proper management of the water cycle involves the application of existing technological knowledge to achieve socially and economically useful objectives, such as the protection of surface and underground of water bodies and the correct management of water resources. The economic advantage of reuse lies in providing the community with a water supply, at least for some uses (for example, in the agricultural field for irrigating the fields) for which high-quality water is not required, at lower costs, as recycling costs less than disposal [5]. In order to be able to reuse water for any purpose, a certain degree of quality must still be achieved. Conventional treatments are almost never enough, and therefore the technology is moving towards the development of new alternative disinfection treatment systems, aimed at obtaining a high degree of water quality, through the abatement of the microbial, nutrient and toxic substances [6,7].

Due to the advantages and future prospects that can be offered by recycling wastewater, new technologies have been created that seek to obtain efficient processes to guarantee a purified water supply at low cost. It is important to note that, due to the scarcity of water resources, many countries allocate purified wastewater for drinking purposes. This practice in countries with very low availability of technology and widespread electricity can be particularly dangerous for human health. Not all wastewater has the same nature and characteristics; therefore, to identify the most suitable depurative treatment system, it is essential to know both the origin of the polluted water and its final destination. Wastewater from human metabolism, and therefore domestic and/or civil waste, has a clear prevalence of organic pollutants, natural but also synthesized as drugs or biologically active substances [8,9,10,11,12,13].

Disinfection is a fundamental phase in water-purification processes. Today, chlorine and chlorine derivatives dominate the wastewater treatment market, used at more than two-thirds of the plants, and they are able to not only effectively neutralize all species of micro-organisms, but also to degrade organic pollutants, in some cases after a double treatment. However, the various chlorine-based chemicals used in water treatment create degradation byproducts (DPs) that can harm the environment and human health [10,11,12,13]. Tightening regulations on disinfection byproducts and residual chlorine are driving up costs for wastewater plants using those workhorse chemistries. Various studies have therefore been carried out to search for alternative disinfectants, and in recent years peracetic acid has proved to be a viable solution, capable of effectively neutralizing all species of micro-organisms, promoting their use both in the medical field and in industry food. Peracetic acid or peroxyacetic acid (PAA) is the peroxide of acetic acid (AA), considered a strong oxidant and disinfectant, with an oxidation potential larger than that of chlorine or chlorine dioxide. It is a potent antimicrobial agent, more so than water peroxide, rapidly active at low concentrations against a wide spectrum of microorganisms [14]. In many industrial applications PAA solutions are used at around 12%, less unstable than those above 20% that can be potentially explosive, with health hazards similar to those found using 50% hydrogen peroxide. Once a niche product, peracetic acid may soon challenge the dominance of chlorine in large swaths of the wastewater treatment market. The global peracetic acid market was worth $650 million in 2017 and will grow to $1.3 billion by 2026. It is estimated to grow by 8% per year over that same time frame [15]. However, what are its effects on organic pollutants? Are these degraded? Do they give rise to degradation byproducts?

Several studies have shown that PAA produces almost no toxic or mutagenic degradation byproducts from the reaction with organic material present in the treated wastewater [16], if not in some cases mostly carboxylic acids, aldehydes and a few degradation byproducts interacting with amino acids, phenols, and other aromatic substances [17], generally not mutagenic.

The aim of the present study was to analyze the possible use of peracetic acid in wastewater treatment plants, instead of sodium hypochlorite. In particular, caffeine, tramadol, irbesartan, diclofenac and trazodone were considered, chosen because found as emerging pollutants of surface waters and for the first three of which the results obtained in the treatment with sodium hypochlorite are already known [10,12,13], while they are being published for diclofenac. The structures of seven isolated DPs, four deriving from tramadol and two of which were isolated for the first time, two DPs of irbesartan and one of trazodone (the last three also isolated for the first time), have been identified from the crossing of mass spectrometry (MS) and nuclear magnetic resonance (NMR) data and justified by a proposal mechanism of formation. Caffeine and diclofenac were partially degraded, but no DPs were isolated.

## 2. Results and Discussion

### 2.1. Experiments

**Figure 1:**
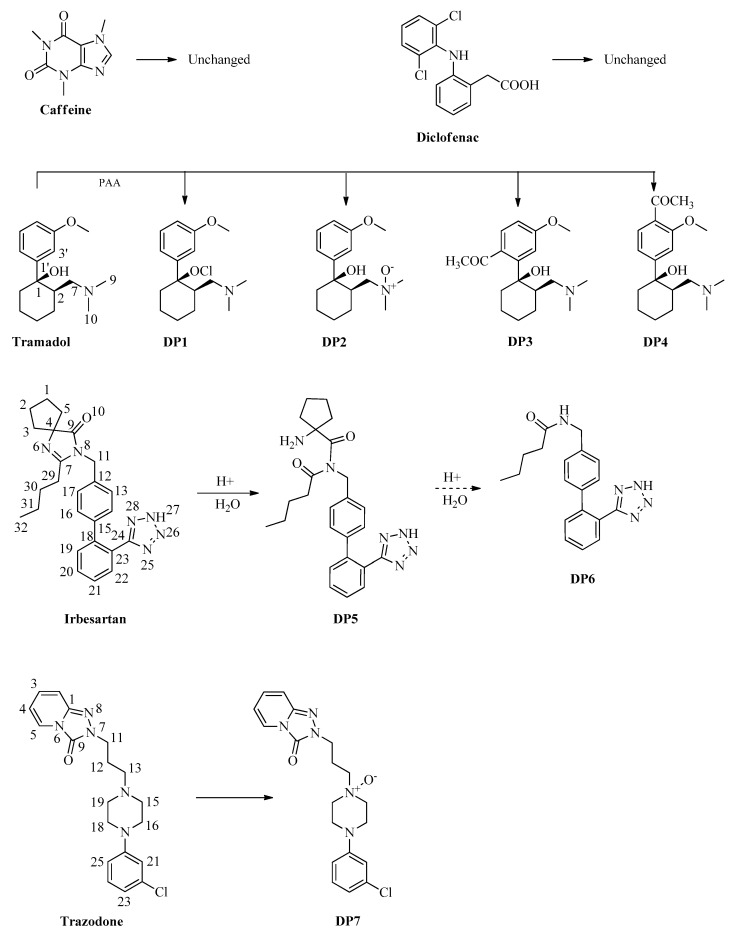
Chemical structures of drugs and their degradation byproducts.

Normally, in degradation studies of an emerging contaminant, a contaminant solution is first taken at very low concentrations (~10^−5^ M), comparable to those detected in surface water or in water entering civil wastewater treatment plants, with a molar ratio of 1:1 contaminant:oxidizing agent [18,19]. Then, the tests are repeated at much higher concentrations of the contaminant (>10^−3^ M), possibly with a much lower ratio of contaminant:oxidizing agent (1:5 or 1:6), so as to be sure of degrading the studied contaminant and then being able to isolate sufficient quantities of degradation byproducts for subsequent spectrometry analyses. In this study, the solution or suspension of the contaminant at concentrations comparable to those in which it was found in the environment appeared substantially unchanged after treatment with peracetic acid. The formation of a few products, which were then isolated and structurally determined, appeared evident only at concentrations of peracetic acid 10 or 100 times higher than that of the contaminant, possibly with long treatment times. Thus, a 10^−5^ M solution of drug was treated for 1 h with 12% peracetic acid (molar ratio drug/peracetic acid, 1:1, with PAA calibrated by using iodometric titration) at room temperature [11,20], simulating the conditions used in a typical wastewater treatment process. The pH of the solution was measured by a pH-meter at 10 min intervals and the course of the reaction was monitored by high-performance liquid chromatography (HPLC) The main degradation byproducts (**DP1**–**DP7**; Figure 1) were identified by comparing their retention times with those of standard compounds commercially available or compounds isolated for the first time. The latter were obtained by performing preparative experiments with a drug solution at a concentration higher than 10^-3^ M treated with 12% peracetic acid, at room temperature. An aliquot of the solution was taken every 10 min, quenched, filtered and fractionated into acidic and neutral fractions, to remove excess of salts and acids present in solution. The course of the reaction was monitored by HPLC. The degradation byproducts obtained were isolated by column chromatography and HPLC and completely characterized using NMR and MS analysis. Finally, **DP1**–**DP7** were isolated at 1.90%, 3.45%, 0.34%, 0.39%, 4.95%, 1.58% and 2.00%, respectively, compared to the initial amount of pollutant considered. The plausible mechanism of their formation from each drug is shown in Figure 1.

### 2.2. Structure Elucidation of Degradation Byproducts **DP1**–**DP7**

The degradation byproducts **DP1**–**DP7** shown in Figure 1 were isolated by chromatography and identified on the basis of their physical features.

**DP1**: white powder. Nuclear magnetic resonance spectra were in accordance with those reported in the literature [10]. The presence of this chlorine byproduct is explained based on the use of tramadol hydrochloride as the starting material.

**DP2**: white powder. It was identified by a comparison of its spectroscopic data (electronic impact/EI mass spectrum, ^1^H- and ^13^C-NMR spectra) with those of authentic standard obtained by synthesis [21]. 

**DP3**: It has the molecular formula C_18_H_27_NO_3_ according to the presence of the molecular ion at *m/z* 305.23 [M]^+^ in the EI mass spectrum and 18 carbon signals in the ^13^C-NMR spectrum. Nuclear magnetic resonance spectra were very similar to the correspondents to tramadol but with few significant differences. The ^1^H-NMR spectrum showed the three aromatic protons H-6’, H-2’ and H-5’, at δ 6.95, 7.21 and 7.36 and correlated, in the heteronuclear single quantum coherence (HSQC) spectrum, to the carbons at δ 117.34, 109.21 and 130.22, respectively. Moreover, the ^1^H-NMR spectrum, in addition to the methoxyl signal at δ 3.97, showed the signal of a singlet integrable for three protons at δ 2.37, correlated in a HSQC spectrum to the signal at δ 30.85, identified in a distortionless enhancement by polarization transfer (DEPT) experiment as a methyl, and the ^13^C-NMR showed the presence of a carbonyl at δ 202.26. The HMBC experiments allowed the assignment of these signals to an aromatic ring 1,2,4-trisubstituted, with a methyl carbonyl bound to C-4’ carbon as well as a methoxyl and a cyclohexane ring bound to the carbons C-3’ and C-1’, respectively.

**DP4**: It has the molecular formula C_18_H_27_NO_3_ according to the presence of the molecular ion at *m/z* 305.21 [M]^+^ in the EI mass spectrum and 18 carbon signals in the ^13^C-NMR spectrum. Nuclear magnetic resonance spectra were very similar to the correspondents to **DP3**. In fact, the ^1^H-NMR spectrum showed the three aromatic protons at δ 6.77, 7.26 and 7.57, correlated in the HSQC spectrum to the carbons at δ 113.98, 132.93 and 114.69, respectively, and identified as the protons H-4’, H-5’ and H-2’. Moreover, the ^1^H-NMR spectrum, in addition to the methoxyl signal at δ 3.84, showed the signal of a singlet integrable for three protons at δ 2.45, correlated in a HSQC spectrum to the signal at δ 30.92, identified in a DEPT experiment as a methyl, and the ^13^C-NMR showed the presence of a carbonyl at δ 206.96. The heteronuclear multiple bond correlation (HMBC) experiments allowed us to assign the structure of this compound as an isomer of the previous one precisely with the methyl carbonyl function linked to carbon C-6’, instead of carbon C-4’, and the methoxyl and the cyclohexane ring linked to the carbons C-3’ and C-1’, respectively.

**DP5**: The EI mass spectrum analysis showed a molecular ion peak at *m/z* 447.26 [M + H]^+^ corresponding to the molecular formula C_25_H_30_N_6_O_2_. The ^1^H-NMR spectrum showed the protons of the methylene CH_2_–11 at δ 4.31. In the HMBC spectrum, these protons were correlated with the signals at δ 142.16 and 131.42, which were identified as the carbons C-12 and C-13/C-17, and with the signals at δ 178.59 and 180.55, which were identified as the carbonyl carbons C-7 and C-9, respectively. The first carbonyl carbon was correlated with the signals at δ 2.41 and 1.50, which were identified as the protons H-29 and H-30 of the alkyl side chain, respectively, whereas the second one was correlated with the signals at δ 2.05 and 2.20, which were identified as the protons H-3 and H-5 of the cyclopentane group, respectively. These correlations support the hydrolysis of the bound C-7/N-6. 

**DP6**: The EI mass spectrum analysis showed a molecular ion peak at *m/z* 336.36 [M + H]^+^ corresponding to the molecular formula C_19_H_21_N_5_O. The ^1^H-NMR spectrum showed the protons of the methylene CH_2_–11 at δ 4.14, which, in the HMBC spectrum, were correlated with the signals at δ 133.34 and 127.48, which were identified as the carbons C-12 and C-13/C-17, and with the signal at δ 171.19, which was identified as the carbonyl carbon C-7. The last one was correlated with the signals at δ 2.06 and 1.53, identified as the protons H-29 and H-30 of the alkyl side chain, respectively. The ^1^H-NMR spectra do not show the signals of the protons of the cyclopentane group, whose carbon was missing in the ^13^C-NMR spectrum, as well as the carbonyl carbon C-9. These correlations support the hydrolysis of the bounds C-7/N-6 and C-9/H-8.

**DP7**: It has the molecular formula C_19_H_22_ClN_5_O_2_ according to the presence of the molecular ion at *m/z* 387.18 [M]^+^ in the EI mass spectrum and 19 carbon signals in the ^13^C-NMR spectrum. Nuclear magnetic resonance spectra were very similar to the correspondents to trazodone. The ^1^H-NMR spectrum showed the four aromatic signals of the pyridine ring at δ 6.51, 7.08, 7.10 and 7.75, correlated in the HSQC spectrum to the carbons at δ 110.80, 115.37, 130.21 and 123.71 and identified as the protons H-3, H-5, H-4 and H-2, respectively, as well as the four aromatic signals of the benzene ring 1,3-disubstituted at δ 6.79, 6.87, 6.89 and 7.18, correlated in the HSQC spectrum to the carbons at δ 114.49, 120.57, 116.58 and 130.30 and identified as the protons H-25, H-23, H-21 and H-24, respectively. The ^1^H-NMR spectra also showed the protons at δ 2.57, 3.44 and 4.19, correlated in the HSQC spectrum to the carbons at δ 22.03, 68.57 and 43.01, respectively, and identified as the protons H-12, H-13 and H-14, as well as the protons H-15/H-19 and H-16/H-18 at δ 3.38 and 3.43, correlated in the HSQC spectrum to the carbons at δ 63.66 and 43.74, respectively.

**Figure 2:**
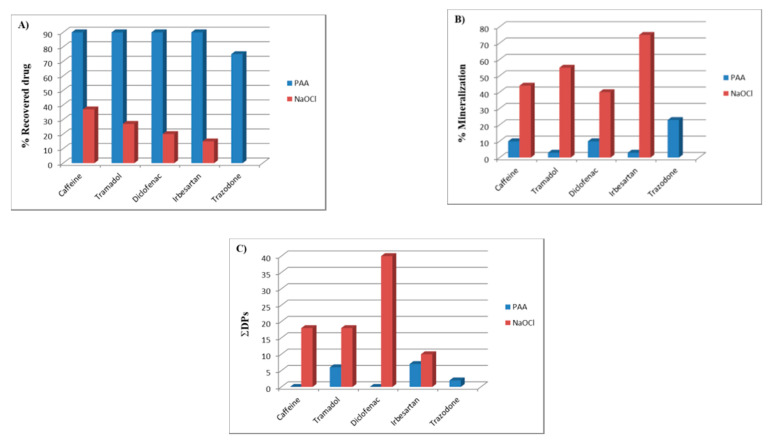
(**A**) Recovery percentages; (**B**) % of mineralization; and (**C**) number of the degradation byproducts of the pollutants considered for treatment with peroxyacetic acid (PAA) and with hypochlorite, respectively.

After treatment with PAA (pollutant:PAA 1:1) and a reaction times of 1 to 4 h, all five considered pollutants were recovered with percentages greater than 90%, and 75% in the case of trazodone only. The percentages were even higher for short reaction times (less than 1 h), and in any case significant if we compare them to the treatment with sodium hypochlorite which was no more than 37% in the case of caffeine, and only 15% in the case of irbesartan. Unfortunately, there are no data in the literature on the degradation of trazodone shown in Figure 2A.

Of course, the mineralization percentage, appraised as percentage by weight, was very low and varied between 10% for caffeine and diclofenac, and 3% for tramadol and irbesartan, with a peak of 23% in the case of trazodone. The corresponding percentage in the case of treatment with hypochlorite was much higher, between 40% and 75%, as shown in Figure 2B. It is interesting to note that in the treatment with PAA, there were four degradation byproducts in the case of tramadol, two for irbesartan and only one for trazodone; no product was isolated in the case of caffeine and diclofenac. However, the transformation percentage of each pollutant ranged between 0% and 7%. In the case of treatment with sodium hypochlorite, there were transformation percentages of 10% for irbesartan, 18% for caffeine and tramadol and 40% for diclofenac, as shown in Figure 2C, with the formation of 6 degradation byproducts for irbesartan and caffeine, 7 for tramadol and 14 for diclofenac.

In the case of tramadol the byproducts **DP1** and **DP2** had already been isolated in the treatment with sodium hypochlorite, whereas **DP3** and **DP4** were isolated for the first time, and it is easy to hypothesize that they derive from an electrophilic substitution on the aromatic ring, to the ortho and para positions of the methoxy group. In particular, five other degradation byproducts were obtained with sodium hypochlorite and were oxidized on the side chain or on the aliphatic ring [10].

In the case of irbesartan, the two degradation byproducts were new products, the first, **DP5**, obtained by hydrolysis of the bond N-6/C-7 and subsequent oxidation of carbon C-7 to the carbonyl group, and the second, **DP6** obtained from **DP5** and by hydrolysis of its amide bond C-9/N-8. By the treatment with sodium hypochlorite, six degradation byproducts were obtained, derived from the hydrolysis of the C-7/N-6 or C-7/N-8 bond of the imidazole-like ring, from the oxidation or loss of the *n*-butyl side chain or from oxidation of benzyl carbon C-11 [13].

The only degradation product obtained from trazodone is the corresponding *N*-oxide (**DP6**) formed on nitrogen N-14 of the piperazine ring, which has been reported by Thummar et al. (2018), by photolytic degradation of trazodone and identified only by mass spectrometry [22].

## 3. Materials and Methods

### 3.1. Drug and Reagents

Caffeine, tramadol, irbesartan, diclofenac and trazodone (99.5%) were purchased from Sigma-Aldrich (Milan, Italy). All the other chemicals and solvents were purchased from Fluka (Saint-Quentin Fallavier, France) with HPLC grade and were used as received. Double distilled water (Microtech, Naples, Italy) was used to prepare the drug solutions.

### 3.2. Reaction 

#### 3.2.1. Apparatus and Equipment

Column chromatography (CC) was carried out on Kieselgel 60 (230–400 mesh, Merck, Darmstadt, Germany). High-performance liquid chromatography was performed on a Shimadzu LC-8A system using a Shimadzu SPD-10A VP UV-VIS detector (Shimadzu, Milan, Italy). Semipreparative HPLC was performed using a RP Gemini C18-110A preparative column (10 μm particle size, 250 mm × 21.2 mm i.d., Phenomenex, Bologna, Italy) with a flow rate of 7.0 mL min^-1^. ^1^H- and ^13^C-NMR spectra were recorded on an NMR spectrometer operating at 400 MHz (Bruker DRX, Bruker Avance, MA, USA), referenced in ppm to residual solvent signals (CDCl_3_, at δ H 7.27 and δ C 77.0) at 25 °C. Proton-detected heteronuclear correlations were measured using a gradient heteronuclear single-quantum coherence (HSQC), optimized for ^1^JHC = 155 Hz, a gradient heteronuclear multiple bond coherence (HMBC), optimized for ^n^JHC = 8 Hz. Electrospray impact mass spectra (ESI-MS) were obtained with a QP-5050A EI 70 eV spectrometer (Shimadzu, Milan, Italy). The MALDI TOF mass spectrometric analyses were performed on a Voyager-De Pro MALDI mass spectrometer (PerSeptive Biosystems, Framingham, MA, USA). 

#### 3.2.2. Peracetic Acid Oxidation Experiments 

As already reported in similar works for the isolation and structural determination of the degradation byproducts of contaminants of surface waters or those ones that arrive in the wastewater treatment plants [11], two tests were performed. In particular, we considered 10^−5^ M solution/suspension of drug treated for 1 h with 12% peracetic acid (molar ratio drug:peracetic acid 1:1) at room temperature, to simulate the conditions used in a typical wastewater treatment process, and preparative experiments with drug concentration at least 100 times higher, to isolate degradation byproducts. The PAA commercial solution was regularly calibrated by using iodometric titration methods [20].

#### 3.2.3. Procedure and Products Isolation

The drug under consideration was dissolved or suspended in double distilled water and treated with peracetic acid. The concentration of the drug, its molar ratio with peracetic acid, the initial and final pH, the duration of the reaction and the percentage of recovery of the starting material are shown in Table 1. The excess of peracetic acid was quenched by excess sodium thiosulfate (0.1 mM, K_2_Cr_2_O_7_ iodometric titration), the pH was brought to neutrality and the solution was concentrated by lyophilization (up to ~50 mL) and extracted with ethyl acetate.

**Tramadol**: The ethyl acetate fraction (EA, 854 mg) was chromatographed on silica gel CC using a gradient of methylene chloride–methanol (99:1 to 10:90, *v*/*v*) to give 15 fractions. The fraction EA6 (62 mg), eluted with methylene chloride–methanol (90:10, *v*/*v*), was analyzed by HPLC using a reversed-phase column and eluting with a gradient of CH_3_COONH_4_ (pH 4.0; 10 mM) and methanol (60:40 to 0:100, *v*/*v*) to give three subfractions. The subfraction EA6.2 contained **DP2** (69 mg) and the subfraction EA6.3 contained **DP1** (38 mg). The fraction EA8 (30 mg), eluted with methylene chloride–methanol (60:40, *v*/*v*), was analyzed by HPLC using a reversed-phase column and eluting with a gradient of CH_3_COONH_4_ (pH 4.0; 10 mM) and methanol (80:20 to 0:100, *v*/*v*) to give three subfractions. The subfraction EA8.2 contained **DP3** (7 mg) and the subfraction and EA8.3 contained **DP4** (7 mg).

**Irbesartan**: The ethyl acetate fraction (EA, 700 mg), obtained by extraction of the aqueous solution at pH = 5, was chromatographed on silica gel CC using a gradient of methylene chloride–methanol (100:0 to 20:80, *v*/*v*) to give seven fractions. The fraction EA2 (60 mg), eluted with methylene chloride–methanol (90:10, *v*/*v*), was analyzed by HPLC using a reversed-phase column and eluting with a gradient of CH_3_COONH_4_ (pH 4.0; 10 mM) and methanol (70:30 to 0:100, *v*/*v*) to give three subfractions. The subfraction EA2.2 contained **DP5** (49 mg). The fraction EA5 (25 mg), eluted with methylene chloride–methanol (75:25, *v*/*v*), was analyzed by HPLC using a reversed-phase column and eluting with a gradient of CH_3_COONH_4_ (pH 4.0; 10 mM) and methanol (70:30 to 0:100, *v*/*v*) to give six subfractions. The subfraction EA5.3 contained **DP6** (16 mg).

**Trazodone**: The ethyl acetate fraction (EA, 625 mg), obtained by extraction of the aqueous solution at pH = 10, was chromatographed on silica gel CC using a gradient of methylene chloride–methanol (100:0 to 20:80, *v*/*v*) to give 15 fractions. The fraction EA12 (96 mg), eluted with methylene chloride–methanol (80:20, *v*/*v*) contained **DP7** (38 mg).

### 3.3. Spectral Data

*2-[(Dimethylamino)methyl]-1-(3-methoxyphenyl)cyclohexyl hypochlorite* (**DP1**). White powder. NMR spectra conform to those recorded for the available standard [10].

*1-((1R,2R)-2-hydroxy-2-(3-methoxyphenyl)cyclohexyl)-N,N-dimethylmethanamine oxide* (**DP2**). White powder. ^1^H-NMR (400 MHz, CDCl_3_): δ 1.47 (m, 1H, H-4), 1.69 (m, 2H, H-5), 1.73 (m, 1H, H-3), 1.77 (m, 1H, H-6), 1.86 (m, 1H, H-4), 2.08 (m, 1H, H-6), 2.33 (m, 1H, H-3), 2.34 (m, 1H, H-2), 2.82 (s, 3H, H-9), 3.18 (s, 3H, H-10), 3.23 (m, 1H, H-7), 3.27 (m, 1H, H-7), 3.83 (s, 3H, OCH_3_), 6.81 (d, *J* = 8.2 Hz, 1H, H-4′), 7.09 (dd, *J* = 8.0, 2.4 Hz, 1H, H-6′), 7.14 (d, *J* = 2.2 Hz, 1H, H-2′), 7.30 (t, *J* = 8.0 Hz, 1H, H-5′). ^13^C-NMR (100 MHz, CDCl_3_): δ 21.55 (C-5), 25.43 (C-4), 30.17 (C-3), 40.53 (C-6), 42.28 (C-2), 55.30 (OCH_3_), 56.20 (C-9), 59.60 (C-10), 72.28 (C-7), 75.45 (C-1), 112.05 (C-4’), 116.68 (C-2’), 117.61 (C-6’), 129.58 (C-5’), 148.55 (C-1’), 159.83 (C-3’). ESI-MS (positive ions): *m*/*z* calculated for C_16_H_25_NO_3_ *m*/*z* 279.18 [M]^+^; found 280.21 [M + H]^+^, 261.11 [M − H_2_O]^+^.

*1-(4-((1R,2R)-2-((dimethylamino)methyl)-1-hydroxycyclohexyl)-2-methoxyphenyl)ethanone* (**DP3**). White powder. ^1^H-NMR (400 MHz, CDCl_3_): δ 1.25 (m, 1H, H-3), 1.40 (m, 2H, H-5), 1.63 (m, 1H, H-3), 1.68 (m, 2H, H-4), 1.90 (m, 1H, H-6), 1.96 (m, 1H, H-6), 2.00 (m, 1H, H-2), 2.13 (s, 6H, H-9/H-10), 2.15 (m, 1H, H-7), 2.29 (m, 1H, H-7), 2.37 (s, 3H, H-8′), 3.97 (s, 3H, OCH_3_), 6.95 (dd, *J* = 8.0, 2.2 Hz, 1H, H-6′), 7.21 (d, *J* = 2.0 Hz, 1H, H-2′), 7.36 (d, *J* = 8.0 Hz, 1H, H-5′). ^13^C-NMR (100 MHz, CDCl_3_): δ 21.37 (C-5), 22.62 (C-3), 24.85 (C-4), 30.85 (C-8’), 40.91 (C-6), 41.34 (C-2), 42.23 (C-9/C-10), 56.32 (OCH_3_), 60.50 (C-7), 75.01 (C-1), 109.21 (C-2’), 117.34 (C-6’), 130.22 (C-4’), 130.22 (C-5’), 146.88 (C-1’), 155.24 (C-3’), 202.26 (C-7’). ESI-MS (positive ions): *m*/*z* calculated for C_18_H_27_NO_3_ *m*/*z* 305.20 [M]^+^; found 305.23 [M]^+^ and 262.23 [M − CH_3_CO]^+^.

*1-(2-((1R,2R)-2-((dimethylamino)methyl)-1-hydroxycyclohexyl)-4-methoxyphenyl)ethanone* (**DP4**). White powder. ^1^H-NMR (400 MHz, CDCl_3_): δ 1.40 (m, 1H, H-3), 1.41 (m, 1H, H-4), 1.60 (m, 1H, H-3), 1.63 (m, 1H, H-4), 1.70 (m, 2H, H-5), 1.92 (m, 2H, H-6), 2.05 (m, 1H, H-2), 2.11 (s, 6H, H-9/H-10), 2.19 (m, 1H, H-7), 2.21 (m, 1H, H-7), 2.45 (s, 3H, H-8′), 3.84 (s, 3H, OCH_3_), 6.77 (dd, *J* = 8.2, 2.4 Hz, 1H, H-4′), 7.26 (d, *J* = 8.2 Hz,1H, H-5′), 7.57 (d, *J* = 2.4 Hz, 1H, H-2′). ^13^C-NMR (100 MHz, CDCl_3_): δ 21.04 (C-5), 21.28 (C-3), 27.09 (C-4), 30.92 (C-8’), 41.35 (C-6), 41.35 (C-9/C-10), 46.02 (C-2), 53.41 (C-7), 55.60 (OCH_3_), 75.90 (C-1), 113.98 (C-4’), 114.69 (C-2’), 120.80 (C-6’), 132.93 (C-5’), 143.39 (C-1’), 158.85 (C-3’), 206.96 (C-7’). ESI-MS (positive ions): *m*/*z* calculated for C_18_H_27_NO_3_ *m*/*z* 305.20 [M]^+^; found 305.21 [M]^+^ and 262.25 [M − CH_3_CO]^+^.

*N-((2’-(1H-tetrazol-5-yl)-[1,1’-biphenyl]-4-yl)methyl)-1-amino-N-pentanoylcyclopentanecarboxamide* (**DP5**). White powder. ^1^H-NMR (400 MHz, CDCl_3_): δ 0.84 (t, *J* = 7.4 Hz, 3H, H-32), 1.29 (sest, *J* = 7.5 Hz, 2H, H-31), 1.50 (q, *J* = 7.5 Hz, 2H, H-30), 1.65 (m, 4H, H-1/H-2), 2.05 (m, 2H, H-3/H-5), 2.20 (m, 2H, H-3/H-5), 2.41 (t, *J* = 7.6 Hz, 2H, H-29), 4.31 (m, 2H, H-11), 6.96 (d, *J* = 8.2 Hz, 2H, H-13/H-17), 7.15 (d, *J* = 8.2 Hz, 1H, H-14/H-16), 7.43 (dd, *J* = 8.2, 2.0 Hz, 1H, H-19), 7.50 (t, *J* = 8.2 Hz, 1H, H-21), 7.58 (t, *J* = 8.2 Hz, 1H, H-20), 7.85 (dd, *J* = 8.2, 2.4 Hz, 1H, H-22). ^13^C-NMR (100 MHz, CDCl_3_): δ 17.56 (C-32), 26.31 (C-31), 28.34 (C-1/C-2), 30.40 (C-30), 37.38 (C-29), 39.32 (C-3/C-5), 47.06 (C-11), 79.31 (C-4), 126.65 (C-23), 131.42 (C-13/C-17), 131.76 (C-19), 132.99 (C-14/C-16), 134.52 (C-22), 134.62 (C-21), 135.19 (C-20), 141.88 (C-18), 142.16 (C-12), 145.44 (C-15), 159.06 (C-24), 178.59 (C-9), 180.55 (C-7). MS-TOF (positive ions): *m*/*z* calculated for C_25_H_30_N_6_O_2_ *m*/*z* 446.24 [M]^+^; found 447.26 [M + H]^+^ (68%). 

*N-((2’-(1H-tetrazol-5-yl)-[1,1’-biphenyl]-4-yl)methyl)pentanamide* (**DP6**). White powder. ^1^H-NMR (400 MHz, CDCl_3_): δ 0.88 (t, *J* = 7.4 Hz, 3H, H-32), 1.34 (sest, *J* = 7.5 Hz, 2H, H-31), 1.53 (q, *J* = 7.5 Hz, 2H, H-30), 2.06 (t, *J* = 7.6 Hz, 2H, H-29), 4.54 (m, 2H, H-11), 7.09 (d, *J* = 8.2 Hz, 2H, H-13/H-17), 7.15 (d, *J* = 8.2 Hz, 1H, H-14/H-16), 7.45 (dd, *J* = 8.3, 2.4 Hz, 1H, H-19), 7.53 (t, *J* = 8.4 Hz, 1H, H-21), 7.60 (t, *J* = 8.4 Hz, 1H, H-20), 7.88 (dd, *J* = 8.4, 2.2 Hz, 1H, H-22). ^13^C-NMR (100 MHz, CDCl_3_): δ 14.21 (C-32), 21.06 (C-31), 26.42 (C-30), 35.74 (C-29), 67.13 (C-11), 124.05 (C-23), 127.48 (C-13/C-17), 128.28 (C-21), 130.18 (C-19), 130.30 (C-14/C-16), 130.76 (C-22), 131.03 (C-20), 133.34 (C-12), 140.41 (C-18), 140.68 (C-15), 156.72 (C-24), 171.19 (C-7). MS-TOF (positive ions): *m*/*z* calculated for C_19_H_21_N_5_O *m*/*z* 335.40 [M]^+^; found 336.36 [M + H]^+^ (83%).

*4-(3-chlorophenyl)-1-(3-(3-oxo-[1,2,4]triazolo[4,3-a]pyridin-2(3H)-yl)propyl)piperazine 1-oxide* (**DP7**). White powder. ^1^H-NMR (400 MHz, CDCl_3_): δ 2.57 (m, 2H, H-12), 3.38 (t, *J* = 11.7, 3.7 Hz, 4H, H-15/19), 3.43 (m, 4H, H-16/18), 3.44 (m, 2H, H-13), 4.19 (t, *J* = 6.3 Hz, 2H, H-11), 6.51 (dt, *J* = 7.4, 1.6 Hz, 1H, H-3), 6.79 (ddd, *J* = 8.3, 2.4, 0.7 Hz, 1H, H-25), 6.87 (ddd, *J* = 7.8, 1.5, 0.6 Hz, 1H, H-23), 6.89 (t, *J* = 1.2 Hz, 1H, H-21), 7.08 (m, 1H, H-5), 7.10 (t, *J* = 8.2, 1.5 Hz, 1H, H-4), 7.18 (t, *J* = 8.2 Hz, 1H, H-24), 7.75 (dd, *J* = 7.1, 1.2 Hz, 1H, H-2). ^13^C-NMR (100 MHz, CDCl_3_): δ 22.03 (C-12), 43.01 (C-11), 43.74 (C-16/18), 63.66 (C-15/19), 68.57 (C-13), 110.80 (C-3), 114.49 (C-25), 115.37 (C-5), 116.58 (C-21), 120.57 (C-23), 123.71 (C-2), 130.21 (C-4), 130.30 (C-24), 135.12 (C-22), 148.65 (C-9), 150.90 (C-20). MS-TOF (positive ions): *m*/*z* calculated for C_19_H_22_ClN_5_O_2_ *m*/*z* 387.15 [M]^+^; found 388.16 [M + H]^+^ (100%).

## 4. Conclusions 

This paper investigated the fate of five emerging pollutants, that is, caffeine, tramadol, irbesartan, diclofenac and trazodone, after treatment with peracetic acid. For all five pollutants, high percentages of the unchanged product were recovered (from 75% to over 90%), even after very long reaction times (from 2 to 4 h), with consequently very low percentages of mineralization (less than 10%) and few degradation byproducts. The best results were obtained with reaction times of 2 h and with a concentration of peracetic acid between 10 and 100 times higher than those of the pollutant to be degraded. A comparison with similar studies on the same drugs, such as the treatment with sodium hypochlorite, indicates that a far higher number of degradation byproducts was obtained, but also higher mineralization percentages (minimum of 40% but with peaks up to more 70%) and consequently minimum recoveries of the products treated (from 10% to 30%). It is therefore necessary to consider that the replacement of sodium hypochlorite (which entails large environmental effects) with peracetic acid may not be beneficial for obtaining the oxidation of the organic material present in wastewater. Additionally, peracetic acid costs almost four times as much as sodium hypochlorite.

## Figures and Tables

**Table 1:** Experimental conditions of the treatments carried out.

Drugs	Drug Conc. (g/L)	Drug/PAA Molar Ratio	pH Initial	pH Final *	Time (h)	% Drug Recovery **
**Caffeine**	1.5	1.0	3.0	6.0	2	>90
	1.5	1.0	3.0	6.0	4	>90
	1.5	0.1	3.0	6.0	1	35
	1.5	0.01	2.5	5.5	1	15
**Tramadol**	2.0	1.0	5.0	6.0	1	>90
	2.0	1.0	5.0	6.0	2	>83
	1.9	0.1	4.0	6.0	1.5	74
	2.0	0.01	1.5	2.5	2	11
**Irbesartan**	2.0	1.0	3.5	5.0	2	>90
	2.0	1.0	3.5	5.0	4	>78
	2.5	0.1	2.0	5.0	2	68
	1.0	0.01	2.5	3.0	2	59
**Diclofenac**	1.5	1.0	3.0	6.0	2	>90
	1.5	1.0	3.0	6.0	4	>77
	1.5	0.1	3.0	6.0	2	35
	1.5	0.01	2.0	6.0	2	25
	1.5	0.01	2.0	6.0	4	19
**Trazodone**	1.5	1.0	5.5	6.0	1	75
	1.5	0.1	2.5	6.0	2	26

* Before the sodium thiosulfate quenching; ** Quantification after fraction collection and byproduct analysis.
